# Localization of Dysenteric Ulcers by X-rays

**Published:** 1918-05

**Authors:** 


***Localization of Dysenteric Ulcers by X-rays.*** By Capt. C. Thurstan Holland, R. A. M. C. (T). Abs. from *Annals of Tropical Medicine and Parasitology*, Feb. 8, 1917.

The author using Glasson's technique for the localization of dysenteric ulcers by X-rays finds that his plates show nothing which is not equally likely to be seen in persons not suffering from dysentery. The writer concludes by asking the question : Why, when it is proved beyond any question that bismuth does not adhere to the surfaces of either gastric or duodenal ulcers, should it adhere to ulcers in the large bowel?

